# High Speed Two-Photon Imaging of Calcium Dynamics in Dendritic Spines: Consequences for Spine Calcium Kinetics and Buffer Capacity

**DOI:** 10.1371/journal.pone.0001073

**Published:** 2007-10-24

**Authors:** L. Niels Cornelisse, Ronald A. J. van Elburg, Rhiannon M. Meredith, Rafael Yuste, Huibert D. Mansvelder

**Affiliations:** 1 Department of Experimental Neurophysiology, Centre for Neurogenomics and Cognitive Research (CNCR), Vrije Universiteit Amsterdam, Amsterdam, The Netherlands; 2 Department of Functional Genomics, Centre for Neurogenomics and Cognitive Research (CNCR), Vrije Universiteit Amsterdam, Amsterdam, The Netherlands; 3 Howard Hughes Medical Institute, Department of Biological Sciences, Columbia University, New York, New York, United States of America; Medical College of Georgia, United States of America

## Abstract

Rapid calcium concentration changes in postsynaptic structures are crucial for synaptic plasticity. Thus far, the determinants of postsynaptic calcium dynamics have been studied predominantly based on the decay kinetics of calcium transients. Calcium rise times in spines in response to single action potentials (AP) are almost never measured due to technical limitations, but they could be crucial for synaptic plasticity. With high-speed, precisely-targeted, two-photon point imaging we measured both calcium rise and decay kinetics in spines and secondary dendrites in neocortical pyramidal neurons. We found that both rise and decay kinetics of changes in calcium-indicator fluorescence are about twice as fast in spines. During AP trains, spine calcium changes follow each AP, but not in dendrites. Apart from the higher surface-to-volume ratio (SVR), we observed that neocortical dendritic spines have a markedly smaller endogenous buffer capacity with respect to their parental dendrites. Calcium influx time course and calcium extrusion rate were both in the same range for spines and dendrites when fitted with a dynamic multi-compartment model that included calcium binding kinetics and diffusion. In a subsequent analysis we used this model to investigate which parameters are critical determinants in spine calcium dynamics. The model confirmed the experimental findings: a higher SVR is not sufficient by itself to explain the faster rise time kinetics in spines, but only when paired with a lower buffer capacity in spines. Simulations at zero calcium-dye conditions show that calmodulin is more efficiently activated in spines, which indicates that spine morphology and buffering conditions in neocortical spines favor synaptic plasticity.

## Introduction

Dendritic spines are tiny protrusions located on dendrites which act as biochemically isolated compartments [1]–[3]. They are the receiving ends of most of the excitatory synapses in the brain. Calcium signaling in these structures attracted much attention in recent years because of its central role in synaptic plasticity. Although synaptic potentiation and depression are both triggered by changes in calcium concentration, they likely require very different concentration profiles [4]–[6]. LTP is reliably triggered by sharp increases in calcium with high magnitude, whereas LTD presumably requires a prolonged modest increase in calcium [5]–[7]. Temporal patterns of pre- and postsynaptic activity may contribute to establishing the different calcium concentration profiles [6]. However, in recent years it has become clear that properties of postsynaptic dendrites and spines are important as well in shaping the kinetics of calcium signaling [2], [3].

Several of these intrinsic properties of spines have been explored experimentally and with computer simulations [1], [2], [8]–[15]. For instance, the presence of calcium buffers slows down kinetics strongly and reduces the magnitude of free calcium increases [2], [8], [15]–[17]. Calcium extrusion by calcium pumps helps to limit the duration of calcium concentration elevation [2], [10], [14]. Many of the inferences on properties of dendritic spines and calcium dynamics have been based on analysis of experimentally measured decay kinetics of calcium signals induced by a single back-propagating action potential. Calcium rise-time kinetics in spines and small dendrites induced with the same protocol are hardly ever addressed experimentally, predominantly due to lack of appropriate time resolution, but it is to be expected that rise time kinetics will be important for peak calcium concentrations that are reached.

Here, we set out to measure both rise and decay kinetics of calcium in neocortical spines and dendrites by parking a two-photon laser specifically on spines and their adjacent dendrites [15], [18]. We addressed the question whether there are differences in how fast calcium rises in spines and dendrites during a back-propagating action potential. We performed additional experiments in combination with computational modeling to investigate what the main determinants are in calcium dynamics in spines and dendrites and how they affect activation of calmodulin, an important protein for synaptic plasticity.

## Results

### Fast calcium dynamics in spines and dendrites

**Figure 1:**
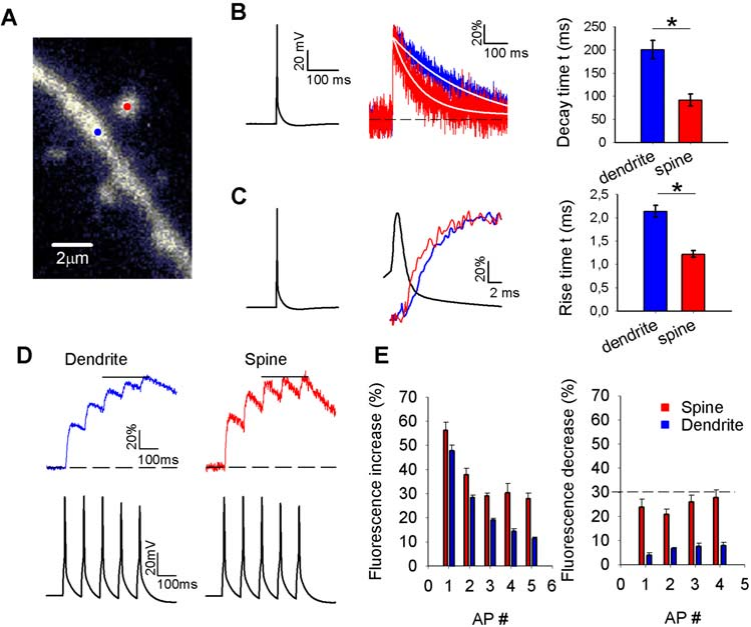
Fast two-photon imaging of calcium rise times in spines and dendrites. A. Image of a targeted spine and dendrite. The laser was successively parked on the spine and dendrite at the sites indicated by the red and blue dot, respectively. B. Fluorescence decay time measurements following a single AP evoked in the soma (left panel) in a dendrite (blue) and spine (red; middle panel). Fluorescence traces were normalized to the peak to facilitate comparison of kinetics between spines and dendrites. White lines represent a mono-exponential fit to the fluorescence decay. Summary data for all fluorescence decay time measurements evoked by a single AP (n = 22 for both spines and dendrites, right panel). Time constants were obtained from mono-exponential fits to fluorescence during the decay phase * p<0.01. C. Same fluorescence changes and AP as in B (left panel), but on a smaller time scale to illustrate differences in rise times of dendrites and spines (middle panel). Traces were again normalized to facilitate comparison. Summary data of all fluorescence rise time measurements evoked by a single AP (n = 22 for both spines and dendrites, right panel) with time constants obtained from mono-exponential fits to fluorescence during the rising phase. *P<0.01. D. Fluorescence changes measured with two-photon point imaging from dendrites (blue) and spines (red) during AP trains. Lower panel: voltage traces with the AP trains induced in the soma. E. Summary data of fluorescence changes during AP trains. Left panel: step sizes induced by individual APs during the 50 Hz train (n = 9). Note that the step sizes continue to decrease in dendrites whereas they remain larger in spines. Right panel: fluorescence decreases after each AP in 50 Hz train. Dotted line indicates the average fluorescence increase induced by the last 3 APs in the train. The decreases in spines almost match these step increases.

To observe calcium dynamics in cortical spines and dendrites, layer 5 pyramidal neurons in visual cortex slices were loaded with the calcium indicator Oregon Green-BAPTA I (100 µM) through a patch pipette. After 20 to 30 minutes whole-cell membrane potential recording, small dendrites and spines were sufficiently labeled and a spine and neighboring dendrite were selected for point imaging (Fig. 1A). We always selected secondary dendrites about 100 µm away from the soma. While imaging continuously from a single location, action potentials (APs) were generated at the soma by injecting current through the recording pipette at 0.5 Hz. Basal fluorescence was carefully monitored during imaging. When basal fluorescence increased more than 10% during imaging, the experiment was excluded from analysis. To improve signal-to-noise ratio on the fluorescence changes during APs, traces were aligned to the AP peak and 20 to 40 APs were averaged (Fig. 1B,C).

Fluorescence changes during APs were rapid in both spines and dendrites but kinetics differed between these compartments (Fig. 1B–D). In line with previous reports [8], [10], [14], fluorescence changes associated with AP firing in dendrites decayed monoexponentially and significantly slower than in spines. The fluorescent signal decayed in dendrites with a time constant of 200.9±19.7 ms (Fig. 1B; P<0.01; n = 22 dendrites in 14 slices; average diameter 1.22±0.06 µm). In spines decays were well fitted by a monoexponential, as was reported for spines connected to thin dendrites [15], and much faster with a time constant of 91.2±12.9 ms (n = 22 spines in 14 slices; average diameter 0.94±0.04 µm). Fluorescence rise times also differed markedly between spines and dendrites (Fig. 1C; P<0.01; n = 22 for both spines and dendrites). In spines, fluorescence increased with a time constant of 1.22±0.07 ms (10% to 90% rise time 3.24±0.16 ms), whereas in dendrites fluorescence rose with a time constant of 2.14±0.12 ms (10% to 90% rise time 4.69±0.27 ms). Note that both in spines and in dendrites the fluorescence signal started to rise during the falling phase of the AP (Fig. 1C). Although the depicted AP was recorded in the soma, the latency of somatic APs traveling over the apical dendrite is about 0.5 ms over the first 200 µm from the soma [19]. Therefore, calcium-induced fluorescence changes in spines and dendrites occur predominantly during the falling phase of the AP, in line with AP-induced calcium influx in presynaptic terminals [20]–[22].

To examine differences in calcium dynamics in dendrites and spines during trains of APs, we applied short trains of five APs at 50 Hz during point imaging from spines and their parent dendrites (Fig. 1D). Fluorescence changes induced by AP trains decayed significantly faster in spines than in dendrites. During AP trains, rise and decay times were also faster in spines than in dendrites, which resulted in a differential fluorescence profile (Fig. 1D). In dendrites, the fluorescence signal continued to build up during the AP train (Fig. 1D,E; n = 9). In contrast, in spines after 2 APs the fluorescence increase during an AP was nearly matched by the following decrease, resulting in little additional overall increase in fluorescence (Fig. 1D,E; n = 9). As a result, fluorescence step sizes per AP during the second half of the train were bigger in spines than in dendrites, indicating that in spines during AP trains *changes* in calcium-bound indicator concentration are bigger than in dendrites.

### Estimating determinants of fast calcium dynamics

**Figure 2:**
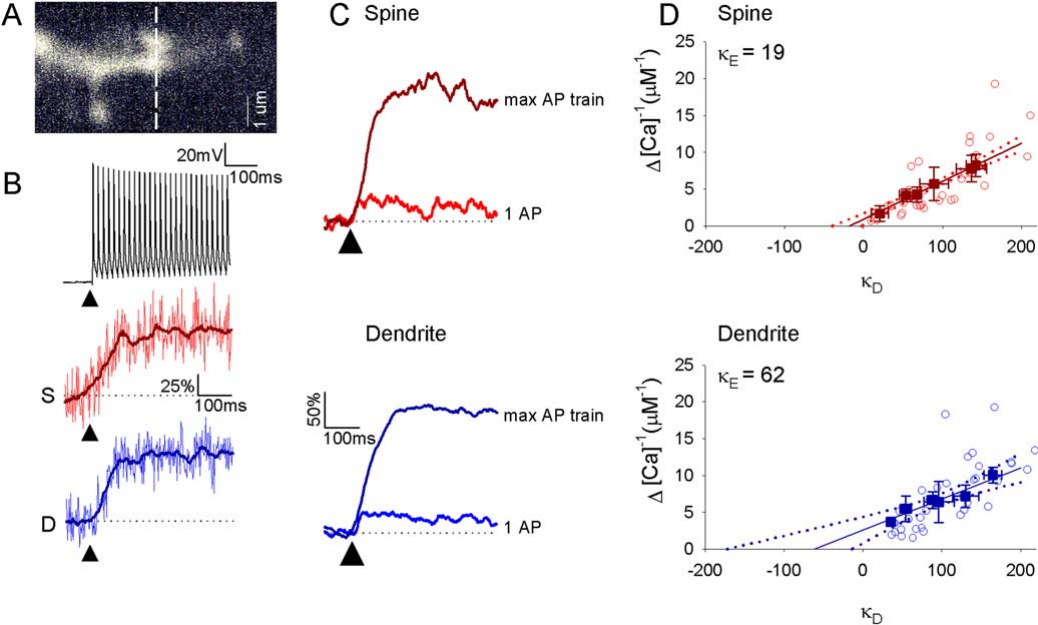
Differing estimates of endogenous buffer capacities in spines and small dendrites. A. Example image of a targeted dendrite and spine pair. Dotted line indicates line-scanned region. B. Back-propagating AP trains were induced to cause maximal dye-saturating calcium indicator fluorescence changes in both spines and dendrites to calculate f_max._ Upper panel, AP train. Lower panels, example maximal fluorescence plateau levels. Darker line indicates boxplot smoothed trace average used for f_max_ calculations. C. Examples of average δf and δf_max_ signals following single AP and AP trains respectively, from spines and dendrites. Arrow indicates onset of stimulation. D. Inverse peak calcium change (Δ[Ca^2+^]_AP_^−1^) following a single AP versus added buffer capacity, κ_D_, for dendrite (blue, upper panel) and spine (red, lower panel). Average values (mean±SEM) for each added buffer concentration (33, 50, 62.5, 75, 90, 100 µM) are plotted over individual data points (open circles) (n = 35). Endogenous buffer capacity (κ_E_) was read off from the intersection of the linear fit with the x axis at zero level of added dye. [Spine κ_E_ = 19 UCI:40, LCI:4; Dendrite κ_E_ = 62 UCI:172, LCI:15). 95% confidence intervals are shown in dotted outlines.

Which determinants of calcium dynamics underlie the differences in calcium signals we observed in spines and dendrites? To answer this question we performed additional experiments using a method developed by Maravall et al. to determine resting calcium levels and the buffer capacity of endogenous calcium buffers in these structures [3], [12]. Although the method is based on a one compartment model that assumes steady state Ca^2+^ binding to both endogenous buffers and dye it yields a good first order estimation of these parameters [16], [23]. Cells were loaded with concentrations of 33, 50, 62.5, 75, 90 and 100 µM OGB-1. Subsequently, line scans were taken from spines and dendrites with 2 ms time resolution (Fig 2A). For the various OGB-1 concentrations calcium changes during a single AP Δ[Ca^2+^]_AP_ was calculated from the fluorescence change during a single AP and the maximal fluorescence during a dye saturating high frequency train of APs (equation 1, Fig 2B,C). Added buffer capacity κ_D_, through loading with the calcium dye, was calculated for the various OGB-1 concentrations using equation 3. The inverse of Δ[Ca^2+^]_AP_ was plotted against κ_D_ in figure 2D in order determine the endogenous buffer capacity κ_E_ by back extrapolation to the horizontal axis crossing using the linear relation between (Δ[Ca^2+^]_AP_)^−1^ and κ_D_ in equation 4 [8], [12]. Dendritic spines had a much lower endogenous buffer capacity compared to dendrites (Fig 2D). Also the 95% confidence interval was smaller in spines (κ_E_ = 19, 95% Upper Confidence Interval: 40, Lower Confidence Interval: 4) than in dendrites (κ_E_ = 62, 95% UCI: 172,LCI:15). From these κ_E_ values, endogenous buffer concentrations were determined, as described in the Methods section. The endogenous buffer concentration in dendrites was 660 µM, whereas in spines it was 210 µM (Table 1). These results suggest that a smaller fraction of entering calcium ions is captured by the endogenous buffer than in dendrites, which will most likely have a strong impact on the free calcium concentration reached during an AP.

**Table 1:** Experimentally obtained parameters.

Parameter	Spine						Dendrite					
	*average*	*SEM*	*n*	*SD*	*min*	*max*	*average*	*SEM*	*n*	*SD*	*min*	*max*
*Measured*												
[Ca^2+^]_0_ (µM)	0.11	0.006	35	0.0355			0.113	0.007	35	0.0414		
Δ[Ca^2+^]_AP_ (µM)	1.05				0.59	7.98	0.383				0.23	1.3
κ_E_	19				4	40	62				15	172
τ_decay_ (ms)	91.2	12.9	22	60.506			200.9	19.7	22	92.401		
τ_rise_ (ms)	1.22	0.07	22	0.3283			2.14	0.12	22	0.5628		
10–90% rise time (ms)	3.24	0.16	22	0.7505			4.69	0.27	22	1.2664		
radius (µm)	0.47	0.02	22	0.0938			0.61	0.03	22	0.1407		
[D]_tot _(µM)	100						100					
*Literature*												
K_D,endo _(µM)	10						10					
K_D,dye _(µM)	0.205						0.205					
N^* ^(µM^−1^ µm^−3^)	602						602					
*Derived*												
SVR factor	3						2					
SVR (µm^−1^)	6.4				6.1	6.7	3.4				3.2	3.6
Δ[Ca^2+^]_tot_ (µM)	21				2.8	161	24				4	96
[B]_end _(µM)	214				44	486	658				159	1827
κ_dye_	48				0	65	92				0	116
n_ions _(µm^−2^)	1981				219	15222	4430				546	17598
γ_0_ (µm/ms)	0.12				0	0.21	0.24				0.01	0.44

### Modeling fast calcium dynamics in spines and dendrites

**Figure 3:**
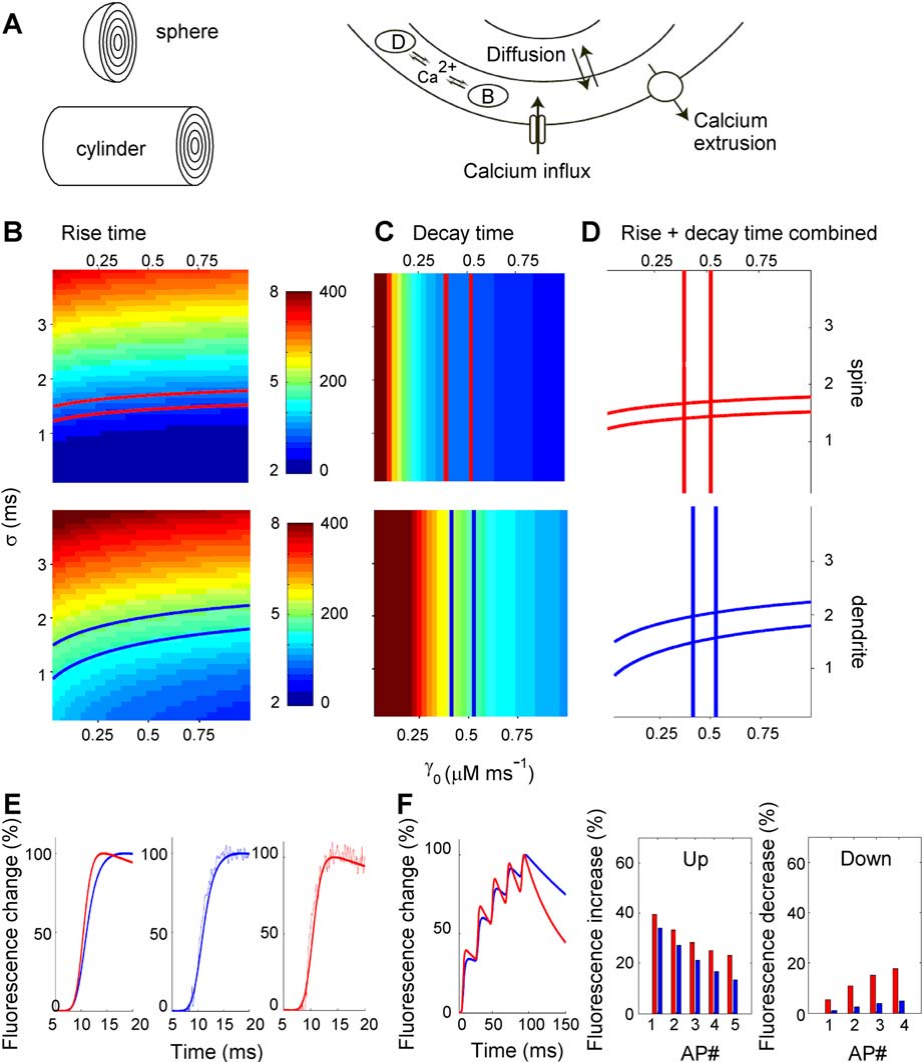
Dynamic modeling of rapid calcium signaling in spines and dendrites. A. Schematic representation of the model components. Spines were modeled as a sphere, dendrites were modeled as a cylinder. Calcium entered and was removed from the outer shell only (shell number 0). Endogenous calcium buffer (B) was fixed while calcium, and calcium indicator dye (D) diffused freely among shells. B, C. Parameter space analysis for the model parameters σ (standard deviation of the Gaussian calcium influx) and γ_0_ (intrinsic extrusion rate). B. Color-coded plot of fluorescence 10% to 90% rise times as a function of σ and γ_0_ for spines (upper panel) and dendrites (lower panel). Simulations were performed for different combinations of σ and γ_0_ with all the other parameters set at their default value (Table 2). Total fluorescence was calculated from the relative contributions of all shells corrected for shell volume. 10–90% rise times were obtained from the total fluorescence signal and plotted in the parameter space for each simulation. Contours indicate the location of fluorescence rise time values obtained in point scan experiments for spines (red) and dendrites (blue) in the parameter space (spine: 3.0–3.4 ms; dendrite: 4.4–5.0 ms). C. Similar plots as in B for fluorescence decay times for spines (upper panel) and dendrites (lower panel). Decay times were fitted from the total fluorescence signal with a mono-exponential. Contours indicate experimental range of decay time constants for spines (red) and dendrites (blue) (spine: 80–100 ms; dendrite: 180–220 ms). Scale bars for color-coding show rise times (left scale) and decay times (right scale) in ms. C. Overlay of contour plots of rise times and decay times showing areas of overlap where the model fits both experimental rise and decay times in spines and dendrites correctly. From these areas the center co-ordinates were extracted for σ and γ_0_ for spines and dendrites that were used as default values in the other simulations in the paper (Table 2). E. Traces of AP-induced fluorescence changes in spines (red) and dendrites (blue) generated by the model plotted on top of representative experimental traces of AP-induced fluorescence changes in spines (middle panel) and dendrites (right panel). F. Traces of calcium bound dye in spines (red) and dendrites (blue) in response to a 50 Hz AP train of 5 APs cf Figure 2E. Quantification of fluorescence step sizes (middle panel) and fluorescence decreases after APs (right panel) during the AP train calculated by the model cf Figure 2F.

To determine free calcium concentration dynamics during non-steady state conditions in the absence of exogenous calcium indicators, we used a dynamic multi-compartmental model of spines and dendrites (Fig 3). It should be emphasized that the calcium dynamics parameters obtained from the buffer capacity experiments above were estimated using a simple one compartment model [3], [12], [16], [23]. This model assumes fast Ca^2+^ equilibration with both endogenous buffers and dye, and does not account for Ca^2+^ diffusion. These assumptions do not hold for the rapidly changing calcium concentrations that occur during and shortly after an AP (Fig 1). Therefore, we used the first order estimations from the buffer capacity experiment as initial parameter settings for the dynamic multi-compartment model for calcium dynamics in small structures to fit fast calcium signals in spines and dendrites. The model served two goals: (1) to investigate which parameters were critical in determining the calcium dynamics in these small structures during non-steady state conditions and (2) to study calcium signaling in unperturbed, calcium indicator-free conditions during non-steady state conditions. In the model, calcium diffusion as well as buffering of calcium by fixed endogenous buffers and diffusible indicator were included and spine and dendrite were respectively modeled as sphere and cylinder (Fig. 3A). Diffusion of calcium between spine and dendrite through the spine neck was not included in the model since it was experimentally shown to be relatively slow (diffusion equilibration time constant ∼90ms, [8]). All model parameters are shown in Table 2 and were derived from the experimental values in Table 1 or obtained from the literature, except for the intrinsic extrusion rate γ_0_ and the time course of the calcium current σ. Parameter σ was experimentally not accessible. The value for γ_0_ was expected to be merely an order of magnitude estimation since it was derived from the relation between the decay time constant and the estimated buffer capacities in the one compartment model (equation 6) which does not take into account that extrusion depends on submembrane calcium concentrations. Therefore, we used the dynamic multi-compartment model to fit the average calcium signals with only σ and γ_0_ as free running parameters.

**Table 2:** Model parameters.

Parameter	Spine	Dendrite	Reference
**Morphology**			
SVR (1/µM)	6.4	3.4	this study
**Calcium influx**			
σ (ms)	1.55	1.75	this study
n_ions_ (µm^−2^)	2000	4400	this study
D_ca_ (µm^2^/ms)	0.22	0.22	based on [46]
**Calcium efflux**			
γ_0_ (µm/ms)	0.46	0.465	this study
[Ca]_0_ (µM)	0.11	0.11	this study
**Dye**			
D_dye_ (µm^2^/ms)	0.05	0.05	[47], [48]
[B]_tot,dye_ (µM)	100	100	pipette concentration
K_D,dye_ (µM)	0.205	0.205	[8]
k_on,dye_ 1/(ms µM)	0.45	0.45	[49]
**Endogenous buffer**			
D_endo_ (µm^2^/ms)	0	0	Immobile endogenous buffer
[B]_tot, endo_ (µM)	210	660	this study
K_D,endo _(µM)	10	10	[50], [23]
k_on,endo_ 1/(ms µM)	0.5	0.5	[50]
**Mobile buffer (Parvalbumin,** **Figure 7** **)**			
D_pv_ (µm^2^/ms)	0.043	0.043	[15]
K_D,pv _(µM)	0.0514	0.0514	[17]
k_on,pv_ 1/(ms µM)	0.019	0.019	[17]
**Mobile buffer (Calbindin,** **Figure 7** **)**			
D_cb_ (µm^2^/ms)	0.043	0.043	[15]
K_D,cb_ (µM)	0.24	0.24	[31]
k_on,cb_ 1/(ms µM)	0.09	0.09	[31]

The fluorescence signals (indicator-bound calcium concentration [CaD] averaged over all shells, taking into account the relative contribution of each shell to the total fluorescent signal) induced by calcium concentration changes during an AP were calculated for different combinations of σ and γ_0_, with σ ranging from 0.1 to 4 ms and γ_0_ ranging from 0.025 to 1 µm ms^−1^. From these traces 10–90% rise times and decay times were fitted and plotted in color-coded plots (Fig. 3B and C). The parameter dependence of rise and decay times was differently oriented in this 2D parameter subspace. The color coded plots showed that the rise-times are critically depending on the time course of the calcium signal and are relatively insensitive to the intrinsic extrusion rate, and vice versa for decay times. From these plots we extracted the contours that showed the experimentally obtained range for rise and decay times for dendrites (blue) and spines (red) (Fig. 3B and C). By plotting these contours on top of each other (Fig. 3D) areas of overlap were obtained in which the model correctly fitted both rise and decay times for fluorescence changes in spines and dendrites. The contour plots show that correct fits for rise and decay times are obtained in spines when σ is in the range of 1.4 to 1.8 ms and γ_0_ in the range of 0.39 to 0.52 µm ms^−1^. For dendrites, fits are correct when σ is in the range of 1.5 to 2.0 ms and γ_0_ in the range of 0.43 to 0.53 µm ms^−1^. Thus, the intrinsic extrusion rate is about 2–4 fold higher, but in the same order of magnitude, as estimated with the one compartment model. The ranges for the extrusion rate in spines and dendrites have a large overlap, which suggests that this parameter is not different between spines and dendrites and cannot explain the faster calcium dynamics in spines. Also the range of standard deviations of the calcium current pulse σ overlapped for spines and dendrites and could therefore not explain the difference in calcium dynamics between spines and dendrites. In subsequent simulations we used the values for σ and γ_0_ as default parameters that gave the best fit for fast calcium dynamics in spines and dendrites (Spines: σ = 1.55 ms and γ_0_ = 0.46 µm ms^−1^; Dendrites: σ = 1.75 ms and γ_0_ = 0.465 µm ms^−1^; Table 2).

In a similar set of parameter space simulations we tested the effect of the number of ions n_ions_, that enter during an AP per unit area membrane on calcium dynamics in spines and dendrites. This parameter was derived from the buffer capacity experiments but spanned a broad range with large overlap in spines and dendrites (Table 1). Rise and decay times were found to be insensitive to n_ions_ when this parameter was varied between 250 to 10000 (data not shown). Therefore n_ions_ could not explain differences in calcium dynamics between spines and dendrites and might be similar in both structures given the broad range of the experimentally determined value (Table 1).

Figure 3E shows simulated indicator fluorescence, OGB-1 [CaD], traces for spines and dendrites and their match with the experimental data. Note that the model traces and the experiments overlap very well, indicating that the model replicates the experiments faithfully. Simulated responses to 5 AP trains also reproduced the experimental data (Fig 3F, left panel). The relative fluorescence increase and decrease in these traces is larger for spines (Figure 3F, right panels) as was found experimentally (Figure 1F). These data suggest that the parameter values in Table 2 give a good description of the parameter settings underlying fast calcium dynamics in spines and dendrites with most likely no or small differences in calcium influx and extrusion parameters between spines and small dendrites.

### Calcium diffusion can not explain faster calcium dynamics in spines

**Figure 4 pone-0001073-g004:**
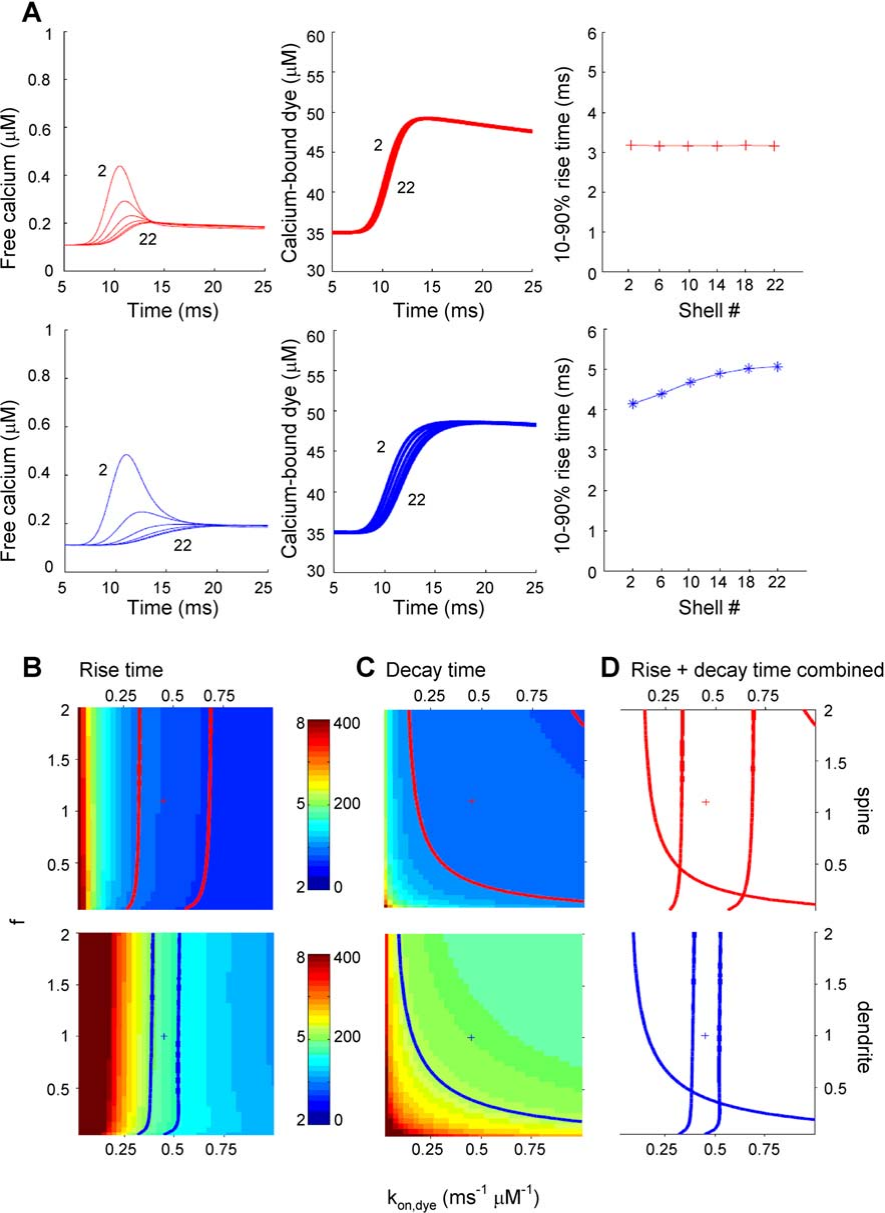
Diffusion cannot explain faster calcium dynamics in spines. (A) Free calcium signals (left panel) and fluorescent signals (calcium bound to dye, middle panel) are plotted for different shells (shell 2, 6, 10, 14, 18, 22) in the multi-compartmental model for spines (upper panels) and dendrites (lower panels). Right panels show 10–90% rise times from the fluorescent signals in the middle panels. B. Parameter space analysis similar as in Figure 3 for model parameters f (diffusion factor) and k_on,dye_ (binding rate of the calcium indicator). B. Color-coded plot of fluorescence 10% to 90% rise times for spines (upper panel) and dendrites (lower panel) with the contours indicating the experimental range for rise time values for spines (red) and dendrites (blue). Plus signs indicate default parameter values for spines (red) and dendrites (blue) as listed in Table 2. C. Similar plots as in B for fluorescence decay times for spines (upper panel) and dendrites (lower panel). Scale bars for color-coding show rise times (left scale) and decay times (right scale) in ms. D. Overlay of contour plots of rise times and decay times showing large areas of overlap where the model fits both experimental rise and decay times in spines and dendrites correctly.

What is the contribution of calcium diffusion to the differences in calcium dynamics between spines and dendrites? During an AP, free calcium diffused strongly in spines (diameter 0.94±0.04 µm) as well as in dendrites (diameter 1.22±0.06 µm; Fig. 4A). The free calcium concentration profile differed strongly between shells. In the outer shell (shell 2) in both spines and dendrites, free calcium concentration increased and fell rapidly within 3 ms and decayed back to baseline with a slow time constant that was dictated by extrusion. In deeper shells, free calcium increased slowly without a concentration overshoot and decayed back to baseline with only the slow time constant. In contrast, in the same simulations the concentration profiles of calcium-bound indicator (OGB-1) showed practically no differences in outer and inner shells. In both spines and dendrites the calcium-bound indicator concentration increased with a similar time course in all shells (Fig. 4A middle and right panels). In spines, rise times did not differ between shells whereas in dendrites the most inner shell (shell 22) was about 1 ms slower than the outer shell (shell 2) and a small delay in the onset of the rise phase of the calcium-bound indicator concentration was present. However, since the rise time of the (fastest) outer shell in the dendrite was already 4.14 ms, only 0.55 ms of the 1.45 ms difference in average rise time between spines (3.24 ms) and dendrites (4.69 ms) could be attributed to diffusion. The strong difference between calcium-bound OGB-1 concentration profile and the free calcium concentration profile (Fig. 4A) suggests that the calcium binding rate of OGB-1 is too slow to compete with fast calcium diffusion. Thus, although free calcium profiles differ strongly between the edge and center of a small dendrite during the rise phase, calcium chelators are too slow to detect these differences.

We investigated how critical the relation between calcium diffusion and buffering is in fast calcium dynamics by conducting a similar analysis in parameter space as was done in Fig 3. Since calcium can diffuse either in the unbound form or bound to the diffusible indicator, we varied the diffusion constant for calcium and dye simultaneously with a multiplication factor f. Rise and decay times are plotted in Fig. 4B–D with f ranging from 0.05 to 2 and k_on,dye_ from 0.025 to 1 ms^−1^ µM^−1^. As expected, binding kinetics of the dye have a strong impact on rise times since faster binding allows the dye to follow the free calcium signal more closely (Fig. 4B–D). On the other hand, diffusion does not have a strong impact on rise and decay times (Fig. 4B–D). In the case of fast diffusion, deeper shells do follow the fast calcium changes closely in the submembrane compartments, apart from the initial overshoot during the rise phase (Fig S1A). In contrast, slow diffusion strongly delays and attenuates calcium signals in deeper shells (Fig S1C). This leads to strong differences in rise times between the shells reported by the dye. However, dye signals in the outer shells dominate the total fluorescence signal and the impact of the slower calcium dynamics in deeper shells is small. This is due to the relatively higher contribution to the total volume of the outer shells compared to the inner ones (49% shell 0–4 and 1% by shell 20–24 for a sphere and 36% shell 0–4 and 6% by shell 20–24 for a cylinder). Altogether, these simulations show that the binding speed of the dye, but not calcium diffusion, is a critical determinant in calcium dynamics in spine and dendrites measured with calcium chelators.

### Lower buffer capacity in spines results in faster calcium dynamics

**Figure 5:**
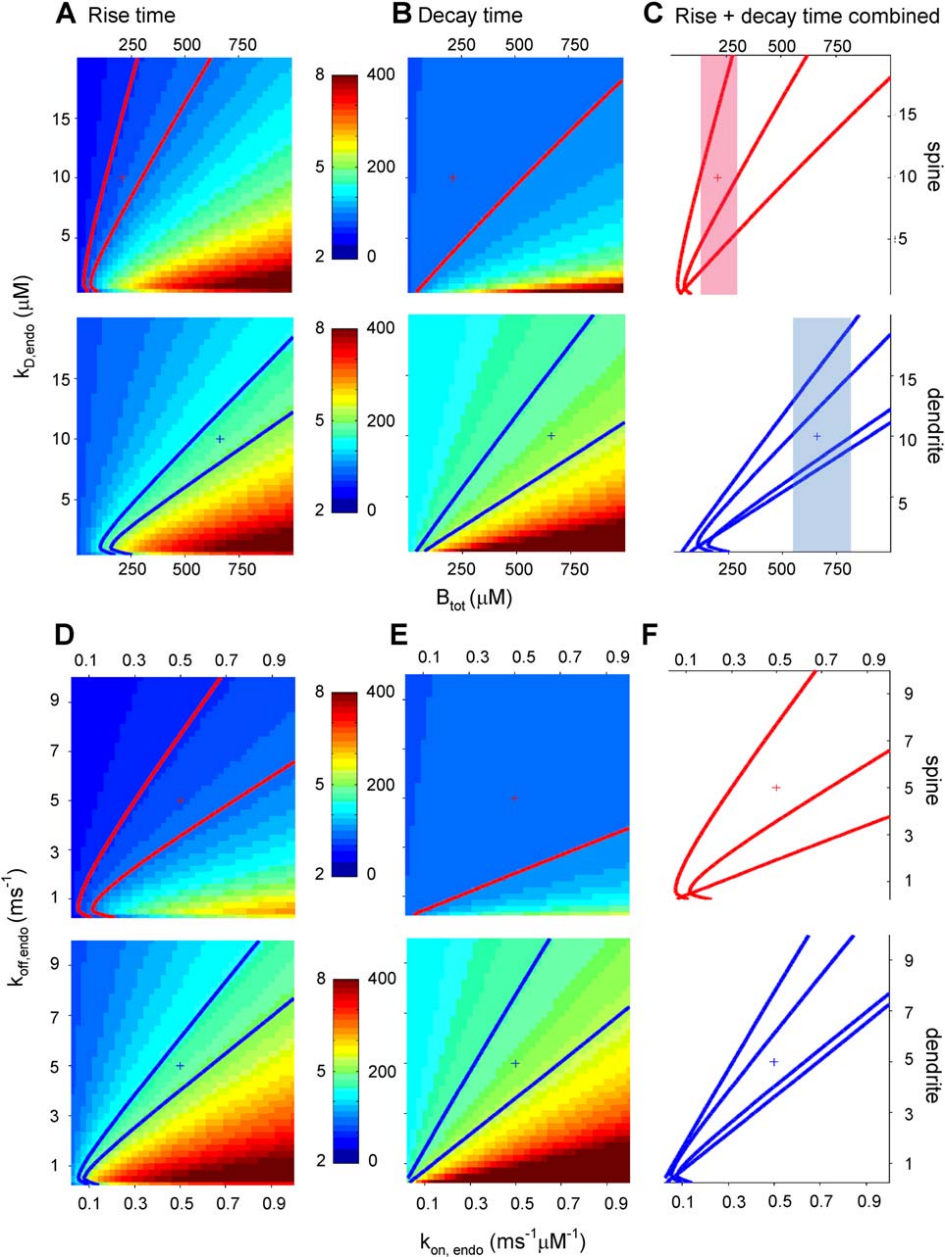
Effect of buffer parameters on calcium dynamics. A–C. Parameter space analysis similar as in Figure 3 and 4 for model parameters K_D,endo_ (dissociation constant for endogenous buffer) and B_tot,endo_ (total concentration endogenous buffer). Plus signs indicate default parameter values for spines (red) and dendrites (blue) as listed in Table 2. C. Overlay of contour plots of rise times and decay times show areas of overlap where the model fits the experimental rise and decay times, with different ranges of B_tot,endo_ for spines (transparent red bar) and dendrites (transparent blue bar). D–F Similar analysis for model parameters k_on,endo_ (binding rate of the endogenous buffer) and k_off, endo_ (unbinding rate of the endogenous buffer). F. Parameter ranges for k_on,endo_ and k_off, endo_ in spines and dendrites display a large overlap.

The buffer capacity experiments indicated that the endogenous buffer capacity in spines is about 3-fold lower than in dendrites. To answer the question if this difference in buffer capacity is a necessary constraint to obtain faster calcium dynamics in spines we tested how critical calcium buffer parameters were in determining rise and decay times. The buffer capacity of a buffer compound depends on the total buffer concentration B_tot_ and the dissociation constant, K_D_ of the buffer (see equation 3) and reduces in a first order approximation to the ratio B_tot_/K_D_. First we varied the dissociation constant K_D_ (0.5–20 µM) and the total buffer concentration B_tot_ (25–1000 µM) for the endogenous buffer (Figure 5A–C). These simulations indicate that indeed the ratio of the buffer parameters B_tot_ and K_D_ is critical in determining the calcium dynamics in spines and dendrites. For a constant ratio of B_tot_ and K_D_ both rise and decay times are fixed. However, especially in dendrites, small changes in B_tot_/K_D_ have a strong impact on rise and decay times (Figure 5A–C). Importantly, the range for B_tot_/K_D_ which yields values for fluorescence rise and decay times in the spine that are in accordance with the fluorescence measurements (i.e. the overlap between the contours for rise and decay times in C) does not overlap with the B_tot_/K_D_ range for dendrites. In other words, no combination of B_tot_ and K_D_ can be found that gives correct rise and decay times for both spines and dendrites in the model. This means that according to the model, assuming the same K_D_ for the endogenous buffer in spines and dendrites, the buffer concentration and hence the buffer capacity has to be set to a lower value in spines compared to dendrites to explain the faster fluorescence transients in spines (Figure 5C, red and blue field). This is in line with the experimental observation in Figure 2. In addition we varied k_on_ and k_off_ , keeping B_tot_ at its default value for spine (210 µM) and dendrite (660 µM, Table 2). Since K_D_ is defined as the ratio k_off_/k_on_ this yielded similar rise and decay times plots (Figure 5D–F) as for K_D_ versus B_tot_ (Figure 5A–C). Again, especially in the dendrite, calcium dynamics were critically dependent on the k_off_/k_on_ ratio but in this case were similar for spines and dendrites. These findings show that also using the dynamic multi-compartmental model a lower endogenous buffer capacity due to a lower endogenous buffer concentration is necessary to explain the faster calcium dynamics in dendritic spines with respect to their parental dendrites.

### Higher surface to volume ratio contributes to faster calcium dynamics in spines

**Figure 6:**
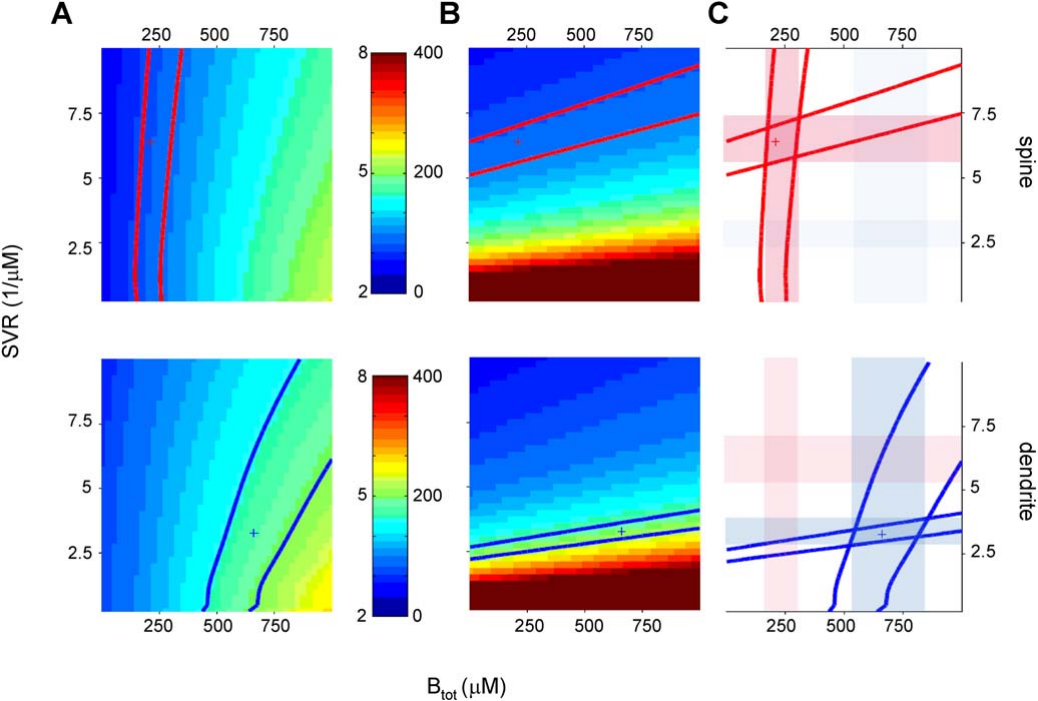
Effect of surface-to-volume ratio and buffer concentration on calcium dynamics. A–C. Parameter space analysis as in Figures 3–5 for model parameters SVR (surface-to-volume ratio) and B_tot,endo_ (total endogenous buffer concentration). C. Parameter ranges for both SVR and B_tot,endo_ differ between spines (red transparent bars) and dendrites (blue transparent bars).

One obvious and experimentally measurable difference between spines and dendrites is the higher surface to volume ratio (SVR) in spines. The spines and dendrites we recorded fluorescence transients from had a diameter of 0.94±0.04 and 1.22±0.06 µm respectively (Fig 1 and 2). However, since calcium influx and extrusion scale with the surface area, SVR is expected to have a significant impact on calcium dynamics. We tested this in Figure 6 where we varied the SVR from 0.25 µm^−1^ to 10 µm^−1^ and B_tot_ from 25–1000 µM to compare their relative contribution to rise and decay kinetics. SVR had a particularly strong impact on decay kinetics, which is in line with the fact that the extrusion rate γ in equation 6 scales linearly with SVR. Rise times were much less affected by SVR. The SVR ranges that yielded good fits for the fluorescence signals in spines and dendrites do not overlap and is particularly narrow for the dendrite, indicating that SVR is a critical parameter for fast calcium dynamics in these structures. As already observed in Figure 5, B_tot_ affects predominantly the rise times and to a lesser extent the decay times, with no overlap between the B_tot_ parameter range for spines and dendrites. Therefore, these simulations clearly indicate that spines shape their fast calcium dynamics by a high surface to volume ratio as well as a lower endogenous buffer capacity.

### Extrapolation to zero exogenous buffering

**Figure 7:**
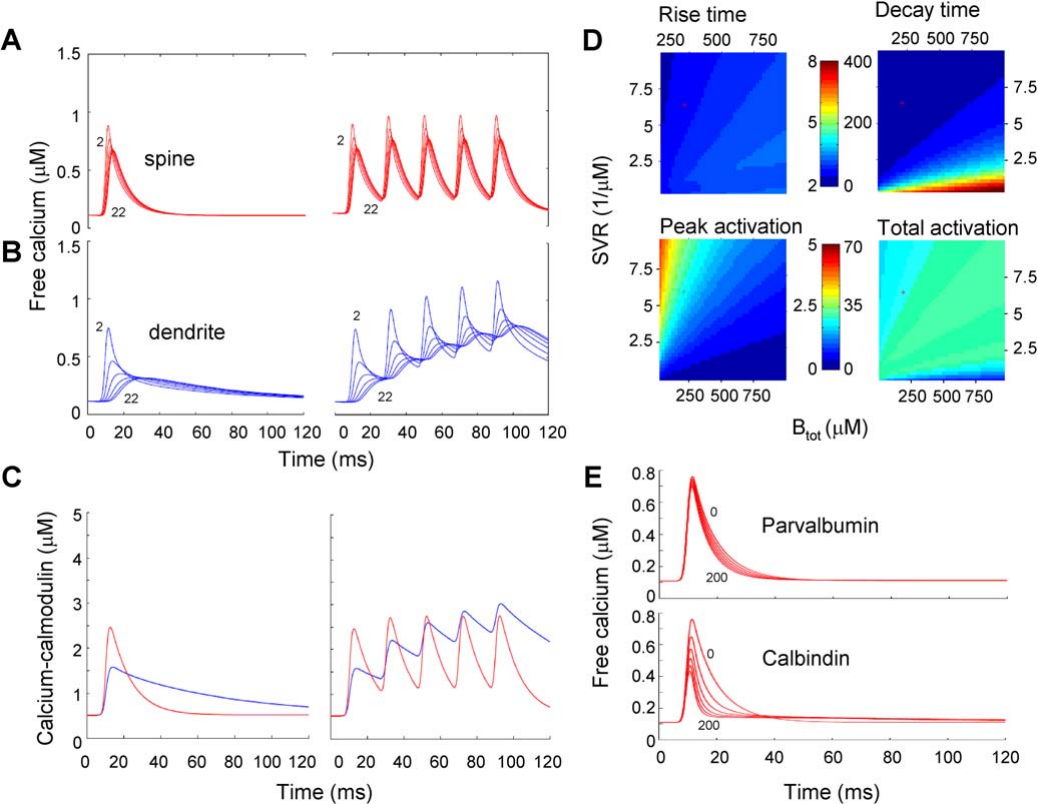
Free calcium dynamics and calmodulin activation during single APs and AP trains. A, B. Model calculations for free calcium dynamics during a single AP (left panels) and during a train of 5 APs at 50 Hz (right panels) in spines (A) and dendrites (B). Plotted are the free calcium concentration traces of shells 2, 6, 10, 14, 18 and 22. C. Calmodulin activation during a single AP and an AP train. Of the total endogenous buffer, 10 µM was assumed to be calmodulin in both dendrites and spines. Shown traces for calcium-bound calmodulin are the total calmodulin signals determined from the relative contributions of all shells corrected for shell volume. D. Parameter space analysis of the effect of model parameters SVR and B_tot,endo_ on calmodulin activation in spines. Upper panels show 10–90% rise time and decay time of the calmodulin signal. Color bar indicates range for rise times (left scale) and decay times (right scale) in ms. Lower left panel shows calmodulin activation at the peak of the signal and lower right panel shows the total calmodulin activation defined as the integral of the calmodulin signal. Color bar indicates range for peak activation in µM (left scale) and total activation in µM ms (right scale). E. Effect of increasing concentrations of the mobile buffers parvalbumin or calbindin on free calcium dynamics in spines. These buffers were added on top of the endogenous buffer concentration. Traces are the average free calcium signals for different mobile buffer concentration obtained from the traces of all shells corrected for shell volume.

By binding calcium, calcium indicators not only report calcium concentration changes, but they perturb these changes as well [23], [24]. To examine profiles of free calcium undisturbed by exogenous calcium indicator, we simulated calcium concentration dynamics with all the parameters at their default setting (Table 2) except for [D]_tot_ which was set to 0 µM. Figure 7A (left panel) shows the free calcium concentration changes in the different shells induced by a single AP. In the absence of calcium indicator, free calcium profiles in spines are very different from free calcium profiles in dendrites (Fig. 7B, left panel). Free calcium reaches much higher concentrations in spines than in dendrites. In addition, calcium kinetics are much faster in all the shells. In spines, free calcium decayed back to baseline within about 50 ms, whereas in dendrites at 50 ms free calcium concentration was still elevated (Fig. 7B). During 50 Hz trains of 5 APs, differences between spines and dendrites in free calcium profiles became more pronounced (Fig. 7A,B, right panels). In spines, free calcium increases were large but returned to baseline with each AP in the train due to the rapid rise and decay kinetics. In contrast, calcium levels in dendrites slowly built up during the AP train. This has implications for calcium signaling in these structures and implies that spines faithfully represent the information encoded in one AP and create a more homogeneous calcium signal across the entire volume, whereas dendrites act more as an integrator of information encoded in a train of APs with a strong gradient in the calcium signal across the radius.

### Effect of different calcium dynamics on calmodulin

Spine and dendritic calcium dynamics are essential for the induction of synaptic plasticity [4]. Calcium/calmodulin-dependent protein kinase II (CaMKII) is most likely the mediator between calcium and induction of long-term potentiation [25]. This kinase is activated by calmodulin, which activates by binding calcium. To examine to what extent the spine and dendritic compartments favor the induction of LTP, we explored how much calmodulin is activated by the free calcium levels predicted by the model. We assumed that 10 µM of the total endogenous buffer concentration was calmodulin [26] both in spines and dendrites. In dendrites about 1 µM calmodulin was activated (Fig. 7C) at the free calcium peak of 0.4 µM induced by a single action potential. In spines, about 2 µM was activated by a free calcium level of 0.7 µM. These levels of activation are in line with quantitative measurements on calcium-dependent calmodulin activation wherein it was found that only at free calcium concentrations of 1 µM calmodulin is half-maximally activated [27]. During a train of action potentials the level of calmodulin activation followed free calcium dynamics closely in spines (Fig. 7C, right panel). In dendrites, calmodulin activation increased, but reached the amount of activation induced in spines only after the third AP. Both in spines and dendrites, the amount of calmodulin activated never increased beyond 30% of the total calmodulin present. This underscores the idea that additional influx of calcium through NMDA receptors is necessary for full activation of calmodulin. However, our simulations do show that spines are better equipped for rapid calmodulin activation and deactivation during high frequency signaling.

Spines display a large variation in their size and shape and endogenous buffer capacity (Fig. 2). To investigate in more detail how these critical parameters for calcium dynamics in spines affect calmodulin activation we performed a similar scan of the B_tot_ and SVR parameter subspace as in Figure 6 but now for the calmodulin signal and in the absence of the calcium dye (Figure 7D). The effect of these parameters is most pronounced for decay and peak of the calmodulin signal. Whereas decay times decrease for spine conditions (low buffer capacity and high SVR) the peak amplitude increases. Since both decay and amplitude determine total calmodulin activation, defined as the integral of the calmodulin signal, this parameter stays relatively constant for different SVR and B_tot_ conditions. Thus, size and buffer capacity of spines determine the calmodulin activation profile but not the total amount of activation.

### Role of endogenous calcium buffers

It is very likely that mobile endogenous calcium buffers were washed out during whole cell recording, even from small compartments such as spines [28]. Diffusible buffers strongly shape free calcium profiles in some cases [29], [30]. We explored how the presence of slow and fast mobile buffers such as parvalbumin and calbindin affect free calcium kinetics in spines. Parvalbumin has a K_D_ of about 50 nM with a k_on_ of 1.9 * 10^7^ M^−1^ s^−1^ [17]. Calbindin binds calcium with two distinct kinetic patterns, of which we included only the fastest binding pattern, k_on_ of 8.7 * 10^7^ M^−1^ s^−1^, with a low K_D_ of about 237 nM [31]. Increasing concentrations of the slow buffer parvalbumin from 0 to 200 µM in spines showed that the peak of free calcium profiles during a single AP was hardly affected (Fig. 7E, upper panel). In the first 30 ms after the AP, when steady state conditions are not reached yet, the decay became increasingly faster with increasing concentrations. This is in line with data obtained on chromaffin cells where free calcium levels also declined more rapidly in the presence of parvalbumin [17]. Rise time kinetics of free calcium were unaffected by parvalbumin. In contrast, increasing concentrations of the faster buffer calbindin from 0 to 200 µM in spines did affect peak levels of free calcium during a single AP (Fig. 7E, lower panel). In addition, the initial decay of free calcium becomes faster but the second decay phase becomes slower with increasing calbindin concentrations. Despite the decline of peak free calcium levels at higher calbindin concentrations, rise times were minimally affected by calbindin. At 0 µM calbindin, 10% to 90% rise time was 2.09 ms, whereas at 200 µM it was 1.98 ms. This was expected from the fact that both mobile buffers are too slow to affect rise-times (see k_on_'s in Table 2 and Figure 5D). Taken together, we conclude that relatively slow calcium buffers such as parvalbumin and calbindin affect free calcium signaling in spines, but that these buffers do not affect the rise time kinetics of free calcium.

## Discussion

### Spines display faster calcium signaling than dendrites

Fast rise times of calcium concentration in dendritic spines associated with single action potentials in pyramidal cells are relevant for processes such as spike-timing-dependent plasticity. In this study we report rise times of calcium-induced fluorescence changes evoked by an action potential in spines and their parent dendrites in neocortex. We find that rise times in spines are about twice as fast as in small dendrites. Similarly, fluorescence decay times are twice as fast in spines as in small dendrites. As a result, during trains of action potentials calcium-induced fluorescence changes remain large in spines, whereas in dendrites overall fluorescence levels increase and changes become smaller rapidly.

We studied fast calcium rise time by parking the two-photon laser on the structure of interest and sampling at 20 kHz the raw PMT signal that collected all emitted light. The advantage of this method over recent advances in fast scanning methods [32] is that in principle the time resolution is limited by filtering frequency and sampling rate. With fast imaging methods, calcium rise times in spines and dendrites have been studied previously in cerebellum and hippocampus [32]–[34]. In cerebellum, these signals were much slower (spine: ∼10 ms; dendrite: ∼15 ms) since they were evoked by a climbing fiber mediated ‘complex spike’, i.e. a large excitatory post synaptic potential (EPSP) with several spikes [34]. In hippocampus, these calcium signals were also much slower, since these were synaptically–evoked [33].

### High SVR as well as low buffer capacity shape fast calcium signals in spines

One obvious difference between spines and dendrites is a larger SVR in spines. Since calcium influx and extrusion scale with the membrane surface, such a larger SVR has a strong impact on the rise and decay phase of the signal. However, we find that in neocortical spines and dendrites with diameters close to 1 µm lower endogenous buffer capacity also contributes to faster calcium dynamics in spines. In addition to SVR, also diffusion and buffering of calcium, influx and extrusion kinetics affect calcium signals. We performed additional experiments to identify the determinants of faster calcium signaling in spines during non-steady state conditions using a dynamic multi-compartment model to fit the experimental calcium traces and to test which parameters are critical for fast calcium dynamics in spines. Previous models describing calcium rise-times in spines were limited, since they did not include diffusion of calcium and dye [10], [15].

The buffer capacity experiments revealed strong variation in buffer capacity between cells with on average a 3-fold lower buffer capacity in spines compared to dendrites. This was confirmed in the model which showed a strong dependence of both rise and decay times on the total buffer concentration and the K_D_ of the endogenous buffer with different ranges for B_tot_ for spines and dendrites. The observed buffer capacity of 19 for neocortical spines was very similar to the endogenous buffer capacity of ∼20 found in hippocampal spines and small dendrites [8]. In small neocortical dendrites we found a 3 fold higher buffer capacity of 62 which is similar to estimates in hippocampal apical dendrites, ∼60 [12], [17], but lower than in cortical apical dendrites, ∼100–200 [16] and dendrites of cortical interneurons, ∼150 [35].

Apart from the SVR, which showed a strong impact on decay times and had distinct parameter ranges in spines and dendrites, other parameters such as calcium current time course, total amount of ions per unit area per AP, intrinsic calcium extrusion rate, K_D_ and binding rates of the endogenous buffer did not differ strongly between spines and dendrites and/or showed overlap in their ranges for spines and dendrites. We conclude that significant differences in calcium dynamics in neocortical spines and dendrites are due to differences in morphology and in endogenous buffer capacity.

### Diffusion can not explain faster calcium dynamics in spines

The multi-compartment model allowed us to investigate the effect of diffusion on calcium signaling. A surprising result of our study is that although free calcium concentration and kinetics differed strongly between shells, depending on their distance from the membrane, the kinetics and concentrations of calcium-bound indicator was very similar across shells (Fig. 4). Even in small dendrites, the rise in calcium-bound indicator concentration was largely independent of distance to the membrane. A similar discrepancy between free calcium concentration profiles and calcium-bound indicator profiles was reported for cerebellar presynaptic terminals [36]. We now show that this also holds for dendritic spines and small dendritic structures. The discrepancy between free calcium and fluorescence signals can be understood from the fact that the binding rate of OGB-1 (0.45 µM^−1^ ms^−1^) is not sufficient to follow rapid calcium increases close to the membrane. Therefore, although the free calcium signals might differ strongly with distance from the membrane, as a result of diffusion these changes are not reported by the dye. Another important reason why diffusion, even in the case of an extremely fast calcium-dye, can not explain differences between calcium kinetics in spines and dendrites can be found in the relative contributions of the different shells. The shells in the submembrane region dominate the overall signal since their volume is relatively large (49% for shells 0–4 in spines) compared to the central shells' volume (1% for shells 20–24 in spines). Altogether, we conclude that diffusion can not explain differences in fast calcium signaling between spines and dendrites.

### Calcium dynamics in physiological conditions

Free calcium dynamics in the absence of indicator dyes in spines as well as in dendrites were fast. In neocortical spines, free calcium signals induced by a single AP rose with time constants that were in accordance with the fluorescence decays measured with low dye concentrations in hippocampus [8]. During a train of 5 APs, free calcium changes in spines are fast enough to follow each individual AP. In contrast, in the dendrite the free calcium level builds up during the train, reaching a similar calcium level after 5 APs as in spines after 1 AP. Therefore, spines faithfully represent the information encoded in an AP train whereas dendrites act as integrators of information encoded in a train of APs.

Adding slow and fast mobile calcium buffers such as parvalbumin and calbindin, which most likely washed out during whole cell recording, even shortens free calcium signals (Fig. 7). This is in line with studies in spiny dendrites of cerebellar Purkinje cells of parvalbumin and parvalbumin/calbindin D_28k_ null-mutant mice [15]. Surprisingly, rise times of free calcium were hardly sensitive to mobile calcium buffers. In the presence of different concentrations of either slow or relatively fast calcium buffers, 10% to 90% rise times varied only 2% in the case of parvalbumin and 12% in the case of calbindin. This is due to the relatively low binding rates of these buffers compared to the endogenous fixed buffer (Table 2). However, peak calcium concentrations were affected by the relatively fast buffers calbindin by up to 50%, suggesting that proteins that rapidly bind calcium will be more responsive to fast calcium signals [37].

### Synaptic plasticity and SVR

Synaptic plasticity depends on calcium signaling. Long-term potentiation (LTP) is induced with brief fast changes in calcium concentration, whereas long-term depression (LTD) is induced by moderate longer lasting calcium increases [4], [5]. In recent years, it is becoming clear that spines of different sizes may reflect different physiological stages with respect to synaptic plasticity [38]. Indeed, it was recently shown that small spines are more likely to contain synapses that undergo long-term potentiation than larger spines. After induction of LTP they increase in size, with volume increases over 50% [39]. Increases in volume, i.e. a decrease of surface to volume ratio, will have great consequences for calcium signaling. In our parameter space analyses, SVR always strongly affected decay times but also rise times. In small spines, calcium will rise much faster and decay much faster. In our simulations we consistently find that with faster rise times, higher free calcium levels are reached. For a spine with a diameter of 0.8 µm that doubles in volume after LTP induction (diameter 1 µm) free calcium during an AP will be reduced by 15%. Therefore, it is likely that with the same AP calmodulin will be activated more in small spines than in large spines (see Figure 7). When comparing small and large spines, small spines will be better tuned for LTP induction. After induction of LTP and size increase [39], calcium signaling will be slower and peak calcium levels will be lower during APs. Less extreme calcium signals might contribute to the stability of large spines observed *in vivo* [40], [41].

## Materials and Methods

### Imaging and electrophysiology

All animal handling and experimentation was done according to NIH guidelines. Coronal slices (300 µm thickness) of visual cortex were prepared from P6-15 C57BL/6 mice, as described [35]. Animals were anaesthetized with ketamine-xylazine (50 and 10 mg kg^−1^). Slices were allowed to recover for at least half an hour before recordings started. All experiments were performed at 33–35° centigrade.

Whole-cell recordings were made using standard electrophysiological methods and equipment. Neurons were filled through the recording pipette with 100 µM Oregon Green-Bapta I alone or in combination with 50 µM Alexa594 (Molecular Probes, Eugene, OR). Pipette solution contained (in mM): 135 KMeSO_4_, 10 KCl, 5 NaCl, 10 HEPES, 2.5 Mg-ATP, 0.3 GTP, pH 7.3 with KOH. After cells were fully loaded with dye (20–30 min after break in), dendritic location or spines were selected for imaging. Imaging was done using a custom-made two-photon laser-scanning microscope [42], [43], consisting of a modified Fluoview (Olympus, Melville, NY) upright confocal microscope with a Ti:Sapphire laser providing 130 fs pulses at 75 MHz at 800–810 nm wavelength (Mira, Coherent, Santa Clara, CA) and pumped by a solid-state source (Verdi, Coherent). A 60×, 0.9 NA water immersion objective (IR1, Olympus) was used. Images were acquired at the highest digital zoom (x10) resulting in a nominal resolution of 30 pixels µm^−1^. To obtain a time resolution well below one millisecond we used point measurements by parking the laser beam. This ‘park mode’ was implemented using in house written software [42], [43]. By calibrating galvanometer command signals the laser beam could be parked on specifically selected structures, such as small dendrites and spines [43]. Laser power was controlled by a Pockels cell (Quantum Technology, Lake Mary, FL) and 5–8mW of laser power was used. The raw PMT signal during point scans was filtered at 5 kHz and digitized at 20 kHz. Rise phase and decay phase were fitted with mono-exponentials. Since 10%–90% rise times could not be determined precisely from all raw fluorescence traces due to noise in some of the experiments, they were obtained from the exponential fit for quantitative comparison with simulated traces.

Endogenous calcium parameters were estimated using a method to measure intracellular calcium concentrations and buffering without wavelength ratioing [8], [12] . Oblique dendrites and spines on secondary dendrites of layer 5 pyramidal cells in visual cortex were located, by addition of Alexa 594 (40 microM) to the intracellular solution (potassium gluconate 140; KCl 1; HEPES 10; K_2_phosphocreatine 4; ATP-Mg 4; GTP 0.4, pH 7.2–7.3, pH adjusted to 7.3 with KOH; 290–300 mOsm) and line-scanned. OGB-1 was used as a calcium indicator with a dynamic range R_f_ of 6 nM and dissociation constant K_D_ of 205 nM [8] at various concentrations (33–100microM). Fluorescence traces were exported into Igor (Igor Wavemetrics, Lake Oswego, OR, USA) for off-line analysis. To determine the maximal fluorescence change [12], trains of 100 action potentials of 62 and 82 Hz were applied (Fig 2B, C). After approximately 100 ms, the fluorescence change reached a plateau, indicating that the calcium indicator reached saturation. We used the highest fluorescence plateaus reached to determine the maximal fluorescence change, which in some cases were from the 62 Hz trains since not all cells could always follow the 82 Hz stimulation reliably. For 1 AP, peak amplitude was measured in the first 100msec after stimulation, with a 10 ms peak around the maximum averaged or a period of 100 ms averaged around the peak following an AP train. An average of 3 single AP traces and 2 AP train traces were combined for each data point. Changes in calcium during 1 action potential or high frequency trains following a baseline of 80 ms f_0_ were reported by the fluorescent signals f and f_max_ respectively. Calcium changes Δ[Ca^2+^] associated with changes in fluorescence from baseline δf≡(f-f_0_)/f_0_ were given by
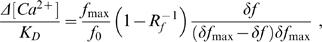
(1)whereas basal calcium [Ca^2+]^_0_ was estimated by
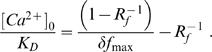
(2)The buffercapacity was defined as the incremental calcium binding ratio
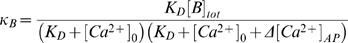
(3)The relation between the calcium change during 1 action potential Δ[Ca^2+^]_AP_ and the total buffercapacity of endogenous buffer and dye (κ_E_+κ_D_)
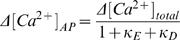
(4)was used to estimate the endogenous buffercapacity κ_E_, with Δ[Ca^2+^]_total_ the change in total calcium (free plus bound) after an action potential. The relationship between (Δ[Ca^2+^]_AP_)^−1^ and κ_D_ was fit by linear regression and extrapolated to the y-axis intercept to obtain Δ[Ca^2+^]_AP_ in the absence of dye and to the x-axis intercept to obtain the endogenous buffercapacity κ_E_ . The number of ions n_ions_ entering the cell per unit area (µm^2^) during an action potential was calculated using the expression
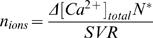
(5)with Δ[Ca^2+^]_total_ calculated with equation 4 for κ_D_ = 0, N^*^ the conversion factor from concentration in µM to number of particles per volume in µm^3^ derived from the Avogadro number, and SVR the surface to volume ratio (SVR = 3/r for spines (sphere) and SVR = 2/r for dendrites (cylinder), with radius r).

In a one compartment model the decay of a calcium signal τ after an action potential is given described by
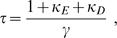
(6)with γ the extrusion rate [16]. We used this expression to get a first order estimation of the intrinsic extrusion rate γ_0_ = γ/SVR (see below) for the multi compartment model using the fitted calcium decay times in spines and dendrites.

Unless mentioned otherwise, two-sided Student t-tests were used, and data are presented as mean±sem.

### Mathematical model

To simulate fast calcium dynamics in spines and dendrites a multi-compartmental shell model was used. In the model, changes in calcium concentration were due to influx through voltage-gated calcium channels induced by a back-propagating action potential, efflux through calcium pumps, radial diffusion and buffering by endogenous buffers and the calcium indicator OGB-1 (Fig. 3A). At physiological temperatures, dendritic APs back-propagate with a velocity of more than 300 µm/ms (i.e. 10 µm is covered in 33 microseconds) [44]. Therefore, calcium influx was assumed to occur uniformly over the dendrite and spine membrane at the imaged site during an AP and only radial diffusion and not longitudinal diffusion was included in the model. Diffusion of calcium between spine and dendrite through the spine neck was not included since it was experimentally shown for spines with monoexponential decay kinetics to be relatively slow (diffusional equilibration time constant ∼90 ms [8], compared to the fast calcium signals in spines and dendrites. To investigate the effect of morphology on calcium dynamics we modeled the spine as a sphere and the dendrite as a cylinder. Local changes in free calcium concentration, [Ca^2+^], were described by
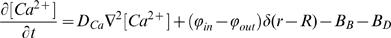
(7)with D_Ca_ the diffusion constant for free calcium, ϕ_in_ and ϕ_out_ calcium influx and efflux over the membrane per unit area (µM µm ms^−1^ ), δ(r-R) the Dirac-delta function, and B_B_ and B_D_ binding of calcium to the endogeneous buffer and calcium dye.

Action potential-induced calcium influx was modeled as a Gaussian-shaped calcium current, as was measured in presynaptic terminals [20]–[22], [37]:
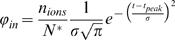
(8)with t_peak_ the time of the peak of the Gaussian calcium current and σ its standard deviation (ms).

Extrusion of calcium was assumed to be linearly dependent on the free calcium concentration,
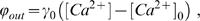
(9)with γ_0_ the intrinsic extrusion parameter (µm ms^−1^), and [Ca^2+^]_0_ the basal calcium level (µM) in spine or dendrite.

In the experiments, imaging started 20 to 30 minutes after establishing the whole cell configuration. Therefore, it is very likely that mobile endogenous calcium buffers were washed out at the start of imaging, even from small compartments such as spines and presynaptic terminals [28]. In the model, mobile endogenous buffers were not included. Since the model had to simulate fast calcium rise times with respect to the relatively slow buffering kinetics, kinetic buffer equations instead of steady state expressions were incorporated. Binding of calcium to the endogenous fixed buffer (B) and the calcium indicator (D) was modeled using the binding reactions
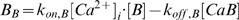
(10)

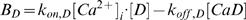
(11)where k_on,B_, k_off,B_ and k_on,D_, k_off,D_ are the on- and off rates for calcium binding to endogenous buffer and calcium dye, [B] and [D] buffer and dye concentration, and [CaB] and [CaD] the concentration of calcium bound to the buffer and dye, respectively. Local changes in fixed buffer concentration are given by
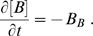
(12)whereas local changes in the diffusible calcium dye are modeled by
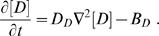
(13)The bound calcium buffer concentrations are given by a local conservation law valid for fixed and initially homogeneously distributed mobile buffers, 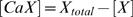 where X is B or D.

In simulations without dye but with calcium-binding proteins Calmodulin, Parvalbumin or Calbindin present (Fig. 7) we used Eq. 11 and 13 to describe buffering kinetics of these endogenous buffers. Simulated traces of the calcium-bound dye concentration ([CaD]) were compared with the experimentally-obtained fluorescence signal.

Numerical simulations were performed in CalC ([45], available from http://web.njit.edu/matveev/calc.html) and analyzed using Matlab (The Mathworks, Natick, MA). The model code and the accompanying analysis code is available from ModelDB http://senselab.med.yale.edu/modeldb via accession number 97903. Rotational and translational symmetries were used to reduce the model to 1 dimension, after which it was put on a grid of 25 points. During the initial phase (20 ms for single action potential, 100 ms for action potential train) simulations were run with a fixed time step of 1 µs, whereas during the decay phase a variable time step was used.

## Supporting Information

Figure S1 Contribution of individual shells to average rise time. A. Free calcium and fluorescence signals in shells 2, 6, 10, 14, 18, 22 of a dendrite in the case of fast binding to the dye (k_on,dye_ = 1) and fast diffusion (f = 2). Binding rate of the dye is too slow to report the overshoot during the rise phase in the free calcium signal but reports calcium signals in the different shells with very similar time courses. B. Parameter space analysis of rise times for model parameters f and k_on,dye_ as in figure 4B lower panel. Rise times in upper right corner and lower right corner of color coded plot in B (white circles) correspond to the weighted average rise times in A and C as indicated with the arrows. C. Free calcium and fluorescence signals in the case of fast binding to the dye (k_on,dye_ = 1) and slow diffusion (f = 0.05) show large differences in rise times between shells. D. Schematic representation of relative contribution of the 5 submembrane shells and the 5 central shells to the weighted average signal of the total dendrite. Shell 0–4 (light blue) dominate the average signal with a contribution of 36% whereas the center shells 20–24 (dark blue) contribute only 6% in case of a cylinder.
(2.32 MB TIF)
