# Supplementary figures and images for: High Speed Two-Photon Imaging of Calcium Dynamics in Dendritic Spines: Consequences for Spine Calcium Kinetics and Buffer Capacity
Source: PLoS One. 2007 Oct 24;2(10):e1073. doi: 10.1371/journal.pone.0001073 (PMC2034355; doi:10.1371/journal.pone.0001073)

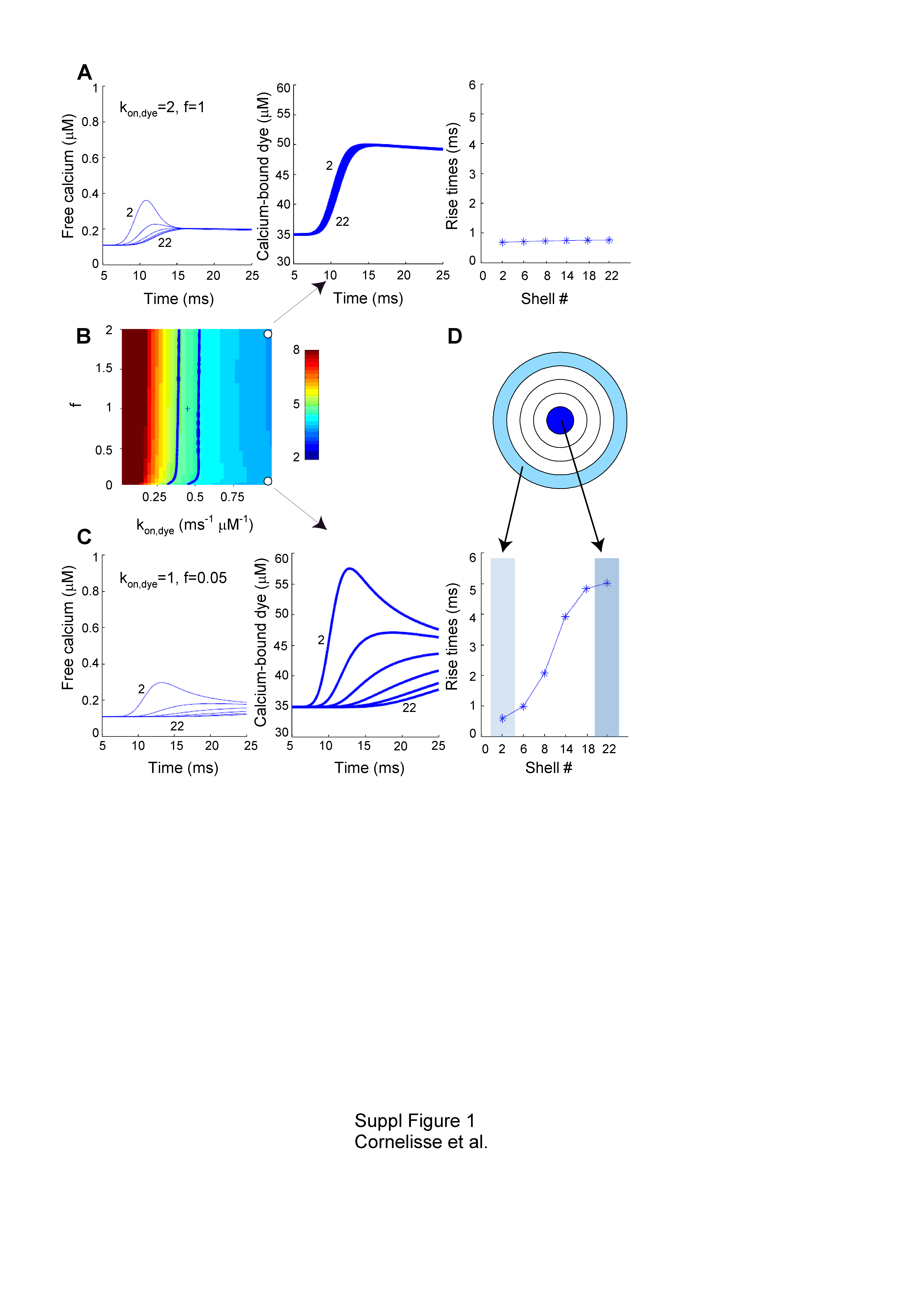

Supplement: Figure S1 — Contribution of individual shells to average rise time. A. Free calcium and fluorescence signals in shells 2, 6, 10, 14, 18, 22 of a dendrite in the case of fast binding to the dye (kon,dye = 1) and fast diffusion (f = 2). Binding rate of the dye is too slow to report the overshoot during the rise phase in the free calcium signal but reports calcium signals in the different shells with very similar time courses. B. Parameter space analysis of rise times for model parameters f and kon,dye as in figure 4B lower panel. Rise times in upper right corner and lower right corner of color coded plot in B (white circles) correspond to the weighted average rise times in A and C as indicated with the arrows. C. Free calcium and fluorescence signals in the case of fast binding to the dye (kon,dye = 1) and slow diffusion (f = 0.05) show large differences in rise times between shells. D. Schematic representation of relative contribution of the 5 submembrane shells and the 5 central shells to the weighted average signal of the total dendrite. Shell 0–4 (light blue) dominate the average signal with a contribution of 36% whereas the center shells 20–24 (dark blue) contribute only 6% in case of a cylinder. (2.32 MB TIF) [file pone.0001073.s001.tif]
